# A Worn-Out Welcome: Renewed Call for a Global Ban on Asbestos

**DOI:** 10.1289/ehp.118-a298

**Published:** 2010-07

**Authors:** Rebecca Clay Haynes

**Affiliations:** **Rebecca Clay Haynes** has written for *EHP *since 1993. Her work has also appeared on National Public Radio and in the *Christian Science Monitor *and *The Environmental Forum*. In addition, she is the author of two children’s science books related to astronomy and space exploration

When Iceland became the first country to ban most forms of asbestos in 1983,^1^ global use hovered around 4.3–4.7 million metric tons per year.^2^ Twenty-five years later, with several dozen countries now implementing some form of ban,^1^ that number had dropped to 2.1 million metric tons.^3^ Today, while demand has plummeted in most developed countries, it continues to rise in those countries now rapidly industrializing. This is the backdrop for the Collegium Ramazzini’s renewal^4^ of its 1999 call^5^ for a global ban on the mining and use of asbestos, which joins a growing chorus from national and international science, health, and labor organizations seeking to ban the mineral worldwide once and for all.

“We’re encouraged by how many countries now ban asbestos, but when you line up those that do not, including the United States, they encompass the majority of the population in the world,” says Joseph LaDou, director of the International Center for Occupational Medicine at the University of California, San Francisco, an emeritus fellow of the Collegium Ramazzini, and lead author of “The Case for a Global Ban on Asbestos” in this issue of *EHP*.^6^ “This problem of asbestos continues to persist where there is the most vulnerable population and the least governmental regulation and enforcement.”

## The Nature of Asbestos

A naturally occurring silicate mineral, asbestos is classified in two forms: amphibole (which includes amosite, crocidolite, tremolite, anthophyllite, and actinolite) and serpentine (also known as chrysotile). Long recognized for its insulating, fire-resisting, and sound-absorbing qualities, asbestos may be mixed with cement or woven into fabric and mats for building construction, vehicle parts, ship-building, and other uses. Chrysotile accounted for 95% of all the asbestos used in the twentieth century^7^ and accounts for nearly 100% of the asbestos still mined and in circulation worldwide.^8^ Amphibole, although rarely mined anymore, is found in vermiculite mined from certain deposits, perhaps most notably the W.R. Grace Mine in Libby, Montana, which closed in 1990.

The asbestos industry argues that chrysotile can be safe with “controlled use,”^9^ but multiple studies^10^ have indicated it is unsafe under any circumstances. The International Agency for Research on Cancer,^11^ U.S. Environmental Protection Agency (EPA),^12^ and National Toxicology Program^13^ have declared all forms of asbestos to be known human carcinogens. In 2006, the International Labour Organization and the World Health Organization both called for asbestos use, including all use of chrysotile, to cease worldwide.^14^,^15^

Microscopic asbestos fibers are easily inhaled and ingested, and the longer and more often one is exposed, the higher the risk for diseases such as asbestosis, lung cancer, and mesothelioma (a rare cancer of the cells lining internal organs, which is believed to develop only with asbestos exposure). Those at greatest risk for exposure and disease include asbestos miners, construction workers, and shipbuilders, followed by family members exposed to residue on workers’ clothing and people living or working near asbestos mines and factories.

An estimated 125 million people continue to be exposed to asbestos in the workplace,^15^ with thousands of deaths and new diagnoses of asbestos-related disease each year.^6^ But many experts believe current death and disease estimates are too low. “Since mesothelioma was not given an International Classification of Diseases code until the mid-1990s, this estimate and other estimates for all countries are probably underestimates,” says Richard Lemen, retired deputy director of the U.S. National Institute for Occupational Safety and Health. “Since many asbestos-related deaths were not coded as such . . . the bottom line is nobody really knows how many such deaths occur each year.” Lemen says he is encouraged that more than 50 countries now ban asbestos but is “greatly discouraged that the U.S. is not in the leadership on this issue.”

Most countries that completely ban the use, extraction, manufacture, and processing of all forms of asbestos began with bans on certain forms or on certain uses such as in schools or ships. “When a country reaches a certain level of economic well-being, it makes sense to ban asbestos,” says environmental consultant Barry Castleman. “They realize there are alternatives, and they don’t need to risk public health. That said, global consumption is now edging back up because countries such as India and China, which have little or no government regulation and protection for workers, are using more of it.”

## Asbestos Abroad

The world’s largest producers of asbestos include Russia, China, Brazil, Kazakhstan, and Canada;^3^ top users are China, Russia, India, Kazakhstan, and Brazil.^16^ The battle over asbestos has occasionally pitted otherwise friendly countries against each other. In 1997, when France banned all forms and uses, including chrysotile, the move set off an international dispute with Canada, which was the world’s third-largest producer at the time. Canada took its case to the World Trade Organization, claiming the ban damaged its economic interests and impeded free trade, and that chrysotile posed no danger with controlled use. After three years of debate, the World Trade Organization ruled that chrysotile was indeed dangerous, that claims about the safety of controlled use could not be supported, and that the French ban was legal to protect public health.^17^

“This was a landmark case,” says Laurie Kazan-Allen, editor of the *British Asbestos Newsletter* and coordinator of the International Ban Asbestos Secretariat, which tracks asbestos-related legislation and other activities worldwide. “The successfully defended ban in France then tipped the European Union [EU] to ban asbestos in 2005. From then on, each country seeking to enter the EU must also ban it. Outside of the EU, we’ve also seen bans introduced by governments forced to take action through a buildup of pressure from citizens opposed to the continued use of an acknowledged carcinogen.”

Canada has a long history with asbestos, starting with its first mine in Quebec in 1874.^18^ Approximately 340 miners now work in Canada’s sole remaining open-pit asbestos mine,^19^ and public pressure has nearly eliminated its use nationwide. However, Canada continues to export chrysotile overseas, especially to developing countries, such as India. Earlier this year, the Canadian Public Health Association and the Canadian Cancer Society called for a ban of chrysotile.^20^,^21^

Most of the asbestos India imports from Canada is used to make corrugated asbestos-cement sheets for building construction. “Anti-asbestos activists in India have staged hunger strikes and written to political leaders in Quebec accusing them of racism for allowing asbestos to be shipped to India when it’s no longer used in Canada,” Castleman says. “But the asbestos industry maintains a close relationship with the government in India— and in Canada, I might add—which makes it hard to reach them with a call for a global ban. Frankly, many importing and exporting countries just blow off these attempts to ban asbestos.” Representatives from the Asbestos Information Association and Canada’s Chrysotile Institute declined to comment for this article.

Although India has banned tremolite and amosite, and some states have imposed moratoriums on new mines, the use of chrysotile is still widespread, according to Madhumita Dutta, a member of the Occupational Health and Environment Network of India, a public health advocacy group. “Asbestos is found everywhere in India, from the shanty towns and urban slums to rural homes and even in middle-class households,” Dutta says. “The battle over asbestos is rooted in the politics and economics of a building material that is considered cheap but only because the price doesn’t reflect the cost to human health and the environment. We’re not sure what effect the Ramazzini call for a global ban might have on the government here, but it may at least provide a good advocacy point for us with medical- and health-related agencies.”

In the rebuilding of Japan after World War II, asbestos was widely used in construction and in the manufacture of ammonium sulfate fertilizer to boost rice production.^22^ But public health concerns began to grow, and by the late 1980s newly formed activist groups started lobbying for a ban, according to Kazan-Allen. In 1995, the government prohibited the use of crocidolite and amosite, and in 2004 chrysotile in new buildings also was banned.^22^ That same year, acting in part on media reports about asbestos, mesothelioma victims and their families began to seek information from the asbestos-cement pipe factories where they had worked. That prompted the Kubota Corporation, a major asbestos-cement pipe producer, to publicly admit that hundreds of its employees as well as people living near factories and mines had become sick or died as a result of exposure.^23^ Within days, other asbestos companies followed with similar announcements, and in 2005 the Japanese government announced a complete phase-out of asbestos over the next three years.^1^

“That scandal, known as the ‘Kubota Shock,’ became an overnight sensation and had a great impact on the national government and public,” says Kazan- Allen. “Finally, after victims’ groups had been trying to raise awareness of the asbestos hazard for so long, asbestos- using corporations were not only admitting their guilt but offering to compensate victims, including widows whose husbands had worked in factories and shipyards. The Japanese govern ment also started covering some medical costs and providing payments to family members.” By one estimate, there could be about 100,000 Japanese deaths from mesothelioma in the next 40 years.^24^

## Asbestos in the United States

Although the United States stopped mining and producing asbestos in 2002, the country imported 1,460 metric tons of chrysotile in 2008, mostly from Canada.^3^ An estimated one- to two-thirds of that imported asbestos is used in roofing products,^3^,^25^ but the greater concern, Castleman says, is for continuing imports of asbestos products no longer made in the United States such as cement pipe, brake pads, and gaskets. “Although the amount used here is minuscule compared to twenty or thirty years ago, we still have auto brakes coming in from Mexico and Asia, for example, with asbestos-lined pads,” says Lemen. “The EPA, other government agencies, and some in Congress have tried to ban asbestos over the years, but industry trade associations have argued such a move would negatively affect their business and the economy.”

The EPA and other federal agencies regulate the use of asbestos, and have attempted full and partial bans since the 1970s, but LaDou and others say trial attorneys and insurance companies have had a greater impact on diminishing nationwide use. Over the past four decades, patients with mesothelioma and other diseases have brought hundreds of thousands of lawsuits against companies that use asbestos, and most insurance companies now refuse to insure projects that include any form of asbestos. “The cost to profit margin has basically pushed asbestos out of the United States,” says LaDou. “The litigation business is very active, and actuaries often have better information to determine risk than we have.” Castleman adds that “regulation, public awareness, and liability have all but ended the use of asbestos in the United States.”

Still, Senator Patty Murray (D–WA) and others have tried since 2002 to pass a “Ban Asbestos in America Act,” legislation that, in addition to implementing a full ban on asbestos under the Toxic Substances Control Act, would also provide federal funds for research and for public education on the dangers of asbestos in the home and workplace. This year, trade organizations again succeeded in keeping the bill from coming to a full vote, according to LaDou, by calling for the continuation of a long-standing exemption for products and materials containing less than 1% asbestos, a level of contamination critics say is not acceptable. “That is hardly a ban on asbestos because it would allow products that contain this small but still dangerous amount,” LaDou says.

Public health advocates in the United States continue to push for a complete ban, according to Linda Reinstein, who co-founded the Asbestos Disease Awareness Organization when her husband was diagnosed with mesothelioma in 2003. “The United States and Canada are the last two major industrialized countries, not counting Russia, that haven’t banned asbestos,” she says. “The impact of this lack of a ban affects not only those of us in North America, of course, but also policies in Asia, Africa, South America, and elsewhere. Asbestos is a proven carcinogen, and there are many safe alternatives. It should be banned entirely here in the United States and worldwide.”

Lemen agrees, saying that a “U.S. and Canadian ban would have a major impact worldwide. Whenever we meet with industry representatives or even scientists from, say, Russia and China, they always throw it in our face, asking why they should bother when our own country doesn’t ban it. ‘[Your law-makers] don’t listen to you,’ they say, ‘so why should we?’”

## What Are the Alternatives?

As alternative materials to asbestos, the U.S. Geological Survey lists calcium silicate, carbon fiber, cellulose fiber, ceramic fiber, glass fiber, steel fiber, wollastonite, and several organic fibers, such as aramid, polyethylene, polypropylene, and polytetra-fluoroethylene. 25 Cellulose and synthetic polyvinyl alcohol and polypropylene fibers used in nonasbestos fiber-cement sheets are mostly of such large diameters that they cannot be breathed into the lungs, according to Castleman. But because these and other alternatives tend to cost 10–15% more than asbestos, large-scale buyers in developing countries still often choose asbestos over an alternative even though, as LaDou argues, “the cost of treating and compensating sick people should more than outweigh the extra cost of an asbestos alternative.”

As awareness of the dangers of asbestos spreads worldwide, consumers increasingly seek products made without it. For example, a public health advocacy group in Brazil sets up public education tables in local parks to inform passersby that asbestos kills,” according to Kazan-Allen. “So when consumers learned that rooftop water storage tanks were made of asbestos-cement, they began to demand an alternative. That led manufacturers to start making them with plastic instead, and slowly but surely, the asbestos industry in Brazil began to lose some of its power, and four of Brazil’s states moved to ban [asbestos] within their borders.”

Collecting precise data on illnesses and deaths caused by asbestos exposure has been a challenge around the world, especially in developing countries. Because of the long latency period between asbestos exposure and mesothelioma diagnosis, LaDou says, an average miner in, say, Brazil may die without any clinician ever making the connection that he had worked with asbestos. To help track the incidence of disease, many countries have created mesothelioma registries whose data, they hope, will convince government officials to ban asbestos. “The registries are part of a two-pronged attack,” says LaDou. “On the public awareness side, activists around the world are working to educate physicians and the public. On the clinical side, we now have data beyond anything we had before because more countries have now set up cancer and specifically mesothelioma registries.”

Even though producer nations continue to send asbestos into the poorest parts of the world, LaDou says there’s clearly a momentum toward a global ban, and he has no doubt asbestos will eventually be banned worldwide. “The main influence on govern ments to ban asbestos comes from the public,” he says. “It can often be difficult to get the asbestos story into the media, but we do see the greatest success when the public is informed and acts on the information. As a professional organization, however, the Collegium’s call for a global ban will most likely have its greatest impact on other professional groups around the world. Its effect is one part of the puzzle.”

## Chronology of National Asbestos Bans^1^

**1983** Iceland bans all types of asbestos (with exceptions); updated in 1996.

**1984** Norway bans all types of asbestos (with exceptions); updated in 1991.

**1986** Denmark bans chrysotile (with exceptions).

Malaysia bans crocidolite.

Sweden introduces the first of a series of bans (with exceptions) on various uses of chrysotile.

**1988** Hungary bans amphiboles.

**1989** Switzerland bans crocidolite, amosite, and chrysotile (with exceptions).

Singapore bans raw asbestos.

**1990** Austria bans chrysotile (with exceptions).

**1991** The Netherlands introduces the first of a series of bans (with exceptions) on various uses of chrysotile

**1992** Italy bans chrysotile (with exceptions until 1994).

**1993** Finland bans chrysotile (with exceptions).

Germany bans chrysotile (with exceptions until 2011).

Croatia bans crocidolite and amosite; updated in 2006 to include all types of asbestos, although that decision was overturned six weeks later.

**1994** Brunei implements administrative rules on asbestos.

**1995** Japan bans crocidolite and amosite.

Kuwait bans all types of asbestos.

**1996** France bans chrysotile (with exceptions).

Slovenia bans production of asbestos-cement products.

Bahrain bans all types of asbestos.

**1997** Poland bans the production and use of asbestos products.

Monaco bans the use of asbestos in all building materials.

**1998** Belgium bans chrysotile (with exceptions).

Saudi Arabia bans all types of asbestos. Lithuania restricts asbestos use.

**1999** United Kingdom bans chrysotile (with exceptions).

**2000** Ireland bans chrysotile (with exceptions).

Argentina bans amphiboles; updated in 2001 to ban chrysotile.

**2001** Latvia bans new uses of asbestos; installed asbestos products must be labeled.

The first in an eventual series of Brazilian states ban asbestos.

Chile bans all types of asbestos.

Oman bans amosite and crocidolite; updated in 2008 to include chrysotile.

**2002** Spain bans chrysotile, crocidolite, and amosite.

Luxembourg bans chrysotile, crocidolite, and amosite.

The Slovak Republic bans all types of asbestos.

New Zealand bans the import of raw asbestos (import of asbestos-containing materials and secondhand asbestos products still allowed).

Uruguay bans all types of asbestos.

**2004** Honduras bans all types of asbestos (with exceptions).

South Africa announces a phase-out of chrysotile over the next three to five years.

Japan bans the new use of chrysotile in building and friction materials.

**2005** Bulgaria bans all types of asbestos. Cyprus, the Czech Republic, Estonia, Greece, Hungary, Lithuania, Malta, Portugal, Romania, and Slovakia pledge to prohibit the new use of chrysotile, other forms of asbestos having been banned previously, under European Union rules.

Japan announces a ban on all types of asbestos within three years.

Egypt bans all types of asbestos.

Jordan bans amosite and crocidolite; updated in 2006 to include all types of asbestos.

**2007** New Caledonia bans all types of asbestos.

**2008** South Africa bans all types of asbestos.

Taiwan bans the use of asbestos in construction materials; updated in 2010 to include virtually all remaining uses of asbestos.

**2009** Republic of Korea (South Korea) bans all types of asbestos.

**2010** Qatar “strictly prohibits” the import of asbestos.

## Figures and Tables

**Figure:**
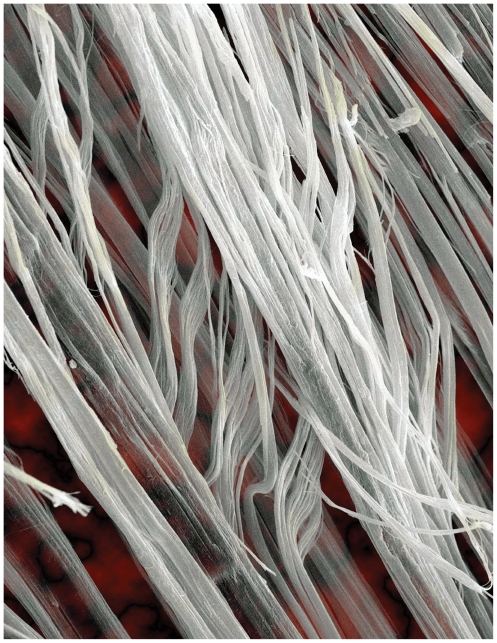
Scanning electron micrograph of chrysotile asbestos fibers

**Figure:**
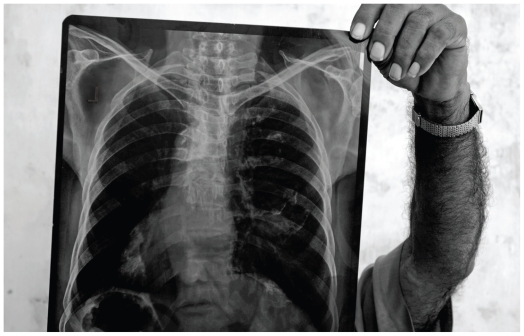
At least eight companies in the Indian state of Gujarat produce asbestos-containing goods. This X ray reveals asbestosis diagnosed in a worker from one of those companies.

**Figure:**
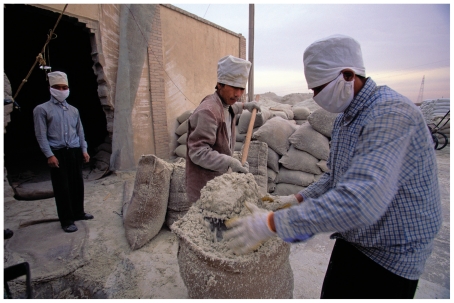
Workers package asbestos in Zhangye, China. In 2008 China produced an estimated 280,000 metric tons of asbestos, making it the world’s second-largest producer. The world leader, Russia, produced an estimated 1,017,000 metric tons in 2008.

**Figure:**
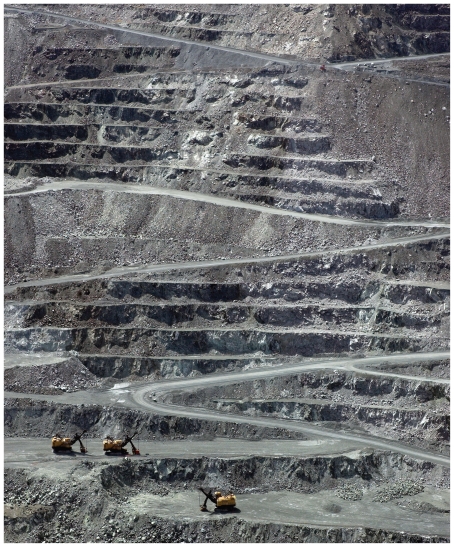
The LAB Chrysotile Mine in Quebec is Canada’s last functional open-pit asbestos mine. At press time, the provincial government was considering a Can$58 million subsidy to complete a new underground mine that could revive Canada’s failing asbestos industry.

**Figure:**
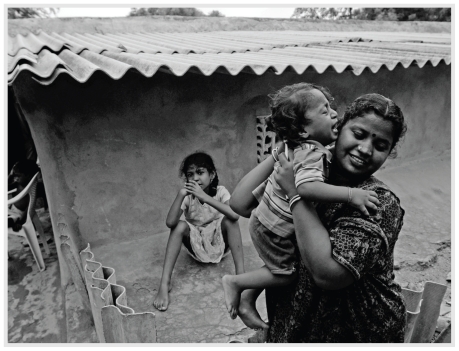


**Figure:**
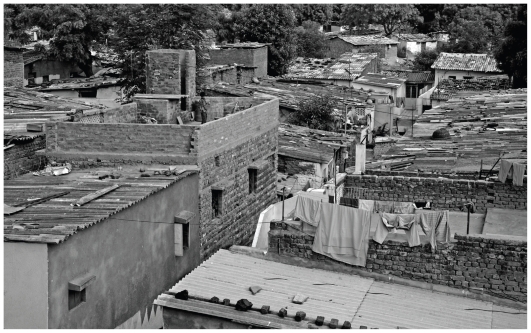
Corrugated asbestos roofing is common in the slums of India (top, Ahmedabad; bottom, New Delhi), where the cheap material is seen as a boon to development. India’s use of asbestos, primarily for roofing, more than doubled between 2000 and 2007. But although asbestos remains safely locked into intact tiles, broken and crumbling roofing can release respirable fibers.
